# Longitudinal Divergence Between CONUT and GNRI Following Decreased Food Intake Temporally Associated With Trimethoprim-Sulfamethoxazole Administration in an Older Patient With Immune Thrombocytopenia and Dementia: A Case Report

**DOI:** 10.7759/cureus.114775

**Published:** 2026-08-19

**Authors:** Takuya Matsunaga, Mariko Watanabe, Kunihiko Ishitani

**Affiliations:** 1 Division of Hematology, Department of Medicine, Higashi Sapporo Hospital, Sapporo, JPN; 2 Department of Nutrition, Higashi Sapporo Hospital, Sapporo, JPN; 3 Division of Internal Medicine, Department of Medicine, Higashi Sapporo Hospital, Sapporo, JPN

**Keywords:** conut, dementia, gnri, immune thrombocytopenia, malnutrition, older adult patient, pneumocystis jirovecii pneumonia, trimethoprim–sulfamethoxazole

## Abstract

Older patients with immune thrombocytopenia are susceptible to infections and treatment-related adverse events, both of which can substantially worsen nutritional status. Although the Controlling Nutritional Status (CONUT) score and the Geriatric Nutritional Risk Index (GNRI) are widely used nutritional screening tools, evidence on their longitudinal changes during complex clinical courses remains limited. An 89-year-old man with vascular dementia was admitted to our hospital with severe thrombocytopenia and bleeding. After treatment with corticosteroids, intravenous immunoglobulin, and eltrombopag, his platelet count recovered. He subsequently developed pneumonia and was clinically diagnosed with probable *Pneumocystis jirovecii* pneumonia based on characteristic chest CT findings and an elevated serum β-D-glucan level, although microbiological confirmation was not obtained. After oral trimethoprim-sulfamethoxazole was initiated, he developed severe appetite loss; although this event was temporally associated with the medication, other contributing factors could not be excluded. His nutritional status consequently deteriorated for a prolonged period and required total parenteral nutrition. Longitudinal evaluation revealed divergent trends between the nutritional indicators. The CONUT score increased from 5 at baseline to 8 during the period of marked nutritional deterioration and subsequently improved to 5 as inflammation resolved. In contrast, the GNRI declined progressively from 81.3 at baseline to 77.4 and then to 72.8, without evidence of recovery, suggesting persistent chronic nutritional risk. This case indicates that, in older patients with dementia, improvement in laboratory-based nutritional indices does not necessarily reflect recovery from chronic malnutrition. Longitudinal evaluation combining the CONUT score and the GNRI may provide complementary information in complex clinical settings.

## Introduction

Immune thrombocytopenia (ITP) is an autoimmune disease characterized by antibody-mediated platelet destruction [1,2]. Older patients with ITP have an increased risk of infection because of age-related immune decline, comorbidities, and immunosuppressive therapy [3-7].

*Pneumocystis jirovecii* pneumonia (PCP) is a major complication of prolonged corticosteroid therapy in non-HIV patients [8]. Trimethoprim-sulfamethoxazole (TMP-SMX) is the first-line treatment for PCP; however, adverse events, including loss of appetite and gastrointestinal symptoms, have been reported [9,10]. In older patients, decreased dietary intake is often multifactorial and may reflect interactions among medication exposure, infection, the hospital environment, and cognitive impairment [11].

The Controlling Nutritional Status (CONUT) score, originally developed by Ignacio de Ulíbarri et al., is an index that reflects acute nutritional and immune status, based on serum albumin, peripheral blood lymphocyte count, and serum total cholesterol levels [12]. In contrast, the Geriatric Nutritional Risk Index (GNRI), proposed by Bouillanne et al., incorporates body weight and reflects chronic nutritional risk [13]. Although both indices have been individually validated, few studies have examined their longitudinal divergence over the course of complex clinical conditions.

CONUT and GNRI assess nutritional status using different clinical and laboratory parameters and may therefore respond differently to changes associated with acute illness and nutritional decline. CONUT incorporates serum albumin, total lymphocyte count, and total cholesterol, and may be affected by inflammatory and metabolic conditions, whereas GNRI is based on serum albumin and the ratio of actual to ideal body weight and may be more closely related to changes in body weight. These differences may be particularly relevant in frail older adults, in whom acute illness, inflammation, reduced dietary intake, and changes in body weight may occur concurrently. However, both indices have limitations and should not be considered definitive measures of nutritional status when interpreted in isolation.

Here, we report an older patient in whom the longitudinal trends of the CONUT score and the GNRI diverged after a decrease in food intake temporally associated with TMP-SMX administration.

## Case presentation

An 89-year-old man with a history of right cerebellar infarction and vascular dementia was transported to our hospital by ambulance on day one, with generalized purpura, epistaxis, oral bleeding, and melena. Blood tests on admission revealed marked thrombocytopenia (platelet count, 1,000/μL) and anemia (hemoglobin, 9.5 g/dL).

The patient was admitted immediately and received platelet and red blood cell transfusions. Given the severity of thrombocytopenia, ITP was suspected, and intravenous prednisolone (50 mg/day) was initiated. Because epistaxis and oral bleeding persisted the following day, intravenous immunoglobulin (IVIG) therapy and oral eltrombopag were added. Hemostasis was subsequently achieved, and oral intake resumed; therefore, corticosteroid therapy was switched from intravenous to oral administration.

Platelet-associated immunoglobulin G (PA-IgG), measured on admission, was markedly elevated (149.2 ng/10⁷ platelets; reference range, <27.6 ng/10⁷ platelets), supporting the diagnosis of ITP [1,2]. Bone marrow examination was not performed due to bleeding risk during the acute phase and the lack of an appropriate time for safe evaluation.

Before admission, the patient required partial assistance with activities of daily living; although his food intake was limited, it had remained stable.

**Figure 1:**
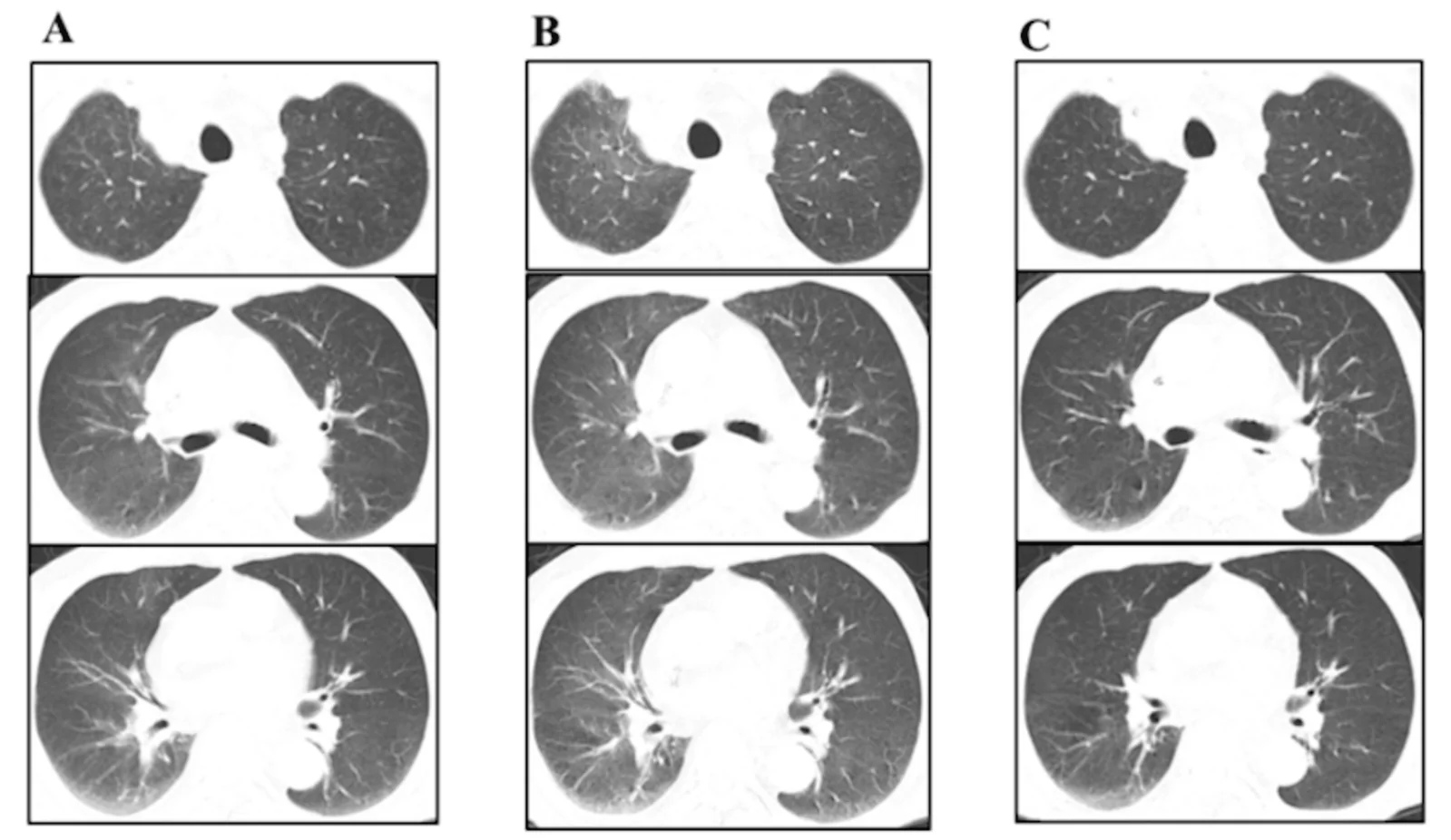
Serial chest computed tomography (CT) findings. (A) CT image obtained on day 67, showing bilateral ground-glass opacities suggestive of *Pneumocystis jirovecii* pneumonia. (B) CT image obtained on day 72, demonstrating progression of the bilateral ground-glass opacities before initiation of trimethoprim-sulfamethoxazole therapy. (C) CT image obtained on day 94, showing marked improvement of the bilateral ground-glass opacities following treatment.

After treatment, the platelet count increased to >200,000/μL and remained at that level. Maintenance therapy with prednisolone 5 mg/day and eltrombopag 12.5 mg/day was continued, and discharge was being considered. On day 67, the serum C-reactive protein (CRP) level increased to 3.13 mg/dL (reference range, ≤0.30 mg/dL). Chest CT showed bilateral ground-glass opacities, raising suspicion of PCP (Figure 1, Panel A). On day 72, the serum β-D-glucan level was elevated (171.6 pg/mL; reference range, ≤20.0 pg/mL), and the bilateral ground-glass opacities on chest CT had worsened (Figure 1, Panel B); therefore, a clinical diagnosis of probable PCP was made [14]. Microbiological confirmation could not be obtained because of the patient’s frailty. Other fungal infections, drug-induced pneumonitis, viral pneumonia, and other opportunistic infections were considered in the differential diagnosis; however, the clinical course supported the diagnosis of PCP.

**Figure 2:**
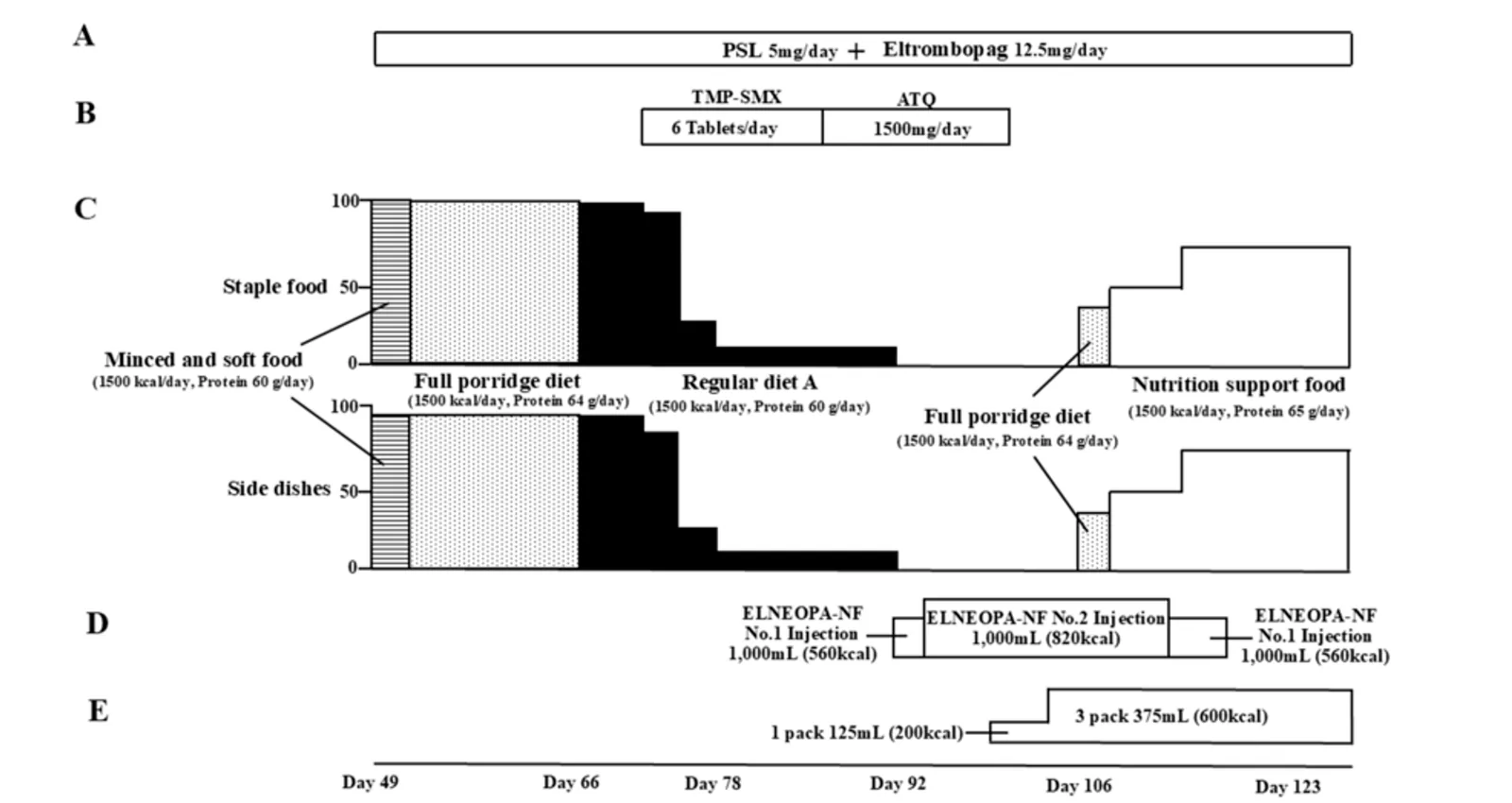
Pharmacologic and nutritional therapies. (A) Oral dosage of prednisolone (PSL) and eltrombopag. (B) Oral dosage of trimethoprim–sulfamethoxazole (TMP-SMX; 80 mg trimethoprim/400 mg sulfamethoxazole per tablet) and atovaquone (ATQ). (C) Hospital meals and intake rates. (D) Total parenteral nutrition. (E) Oral nutritional supplement (Meibalance®). The horizontal axis represents the clinical course using day one as the day of hospital admission. This figure was created using Microsoft PowerPoint (Microsoft Corporation, Redmond, WA, USA).

On day 72, oral TMP-SMX (trimethoprim 80 mg and sulfamethoxazole 400 mg per tablet) was started at six tablets per day (equivalent to TMP 480 mg/day and SMX 2,400 mg/day) (Figure 2, Panels A, B). Chest CT obtained on day 94 showed marked improvement of the bilateral ground-glass opacities (Figure 1, Panel C).

Severe loss of appetite developed shortly after TMP-SMX initiation (Figure 2, Panels B, C). Although this temporal relationship raised the possibility of a TMP-SMX-related adverse effect, causality could not be established. The reduced oral intake may also have been influenced by the underlying infection, systemic inflammation, dementia, hospitalization-related factors, frailty, and concomitant medications.

By day 92, oral intake could no longer be maintained (Figure 2, Panel C). Total parenteral nutrition (TPN) was administered for 28 days, providing 560-820 kcal and 20-30 g of amino acids (equivalent to 20-30 g of protein) per day (Figure 2, Panel D). Subsequently, oral intake gradually improved after the introduction of Meibalance, an oral nutritional supplement (Figure 2, Panel E), and hospital meal consumption increased thereafter (Figure 2, Panel C).

**Figure 3:**
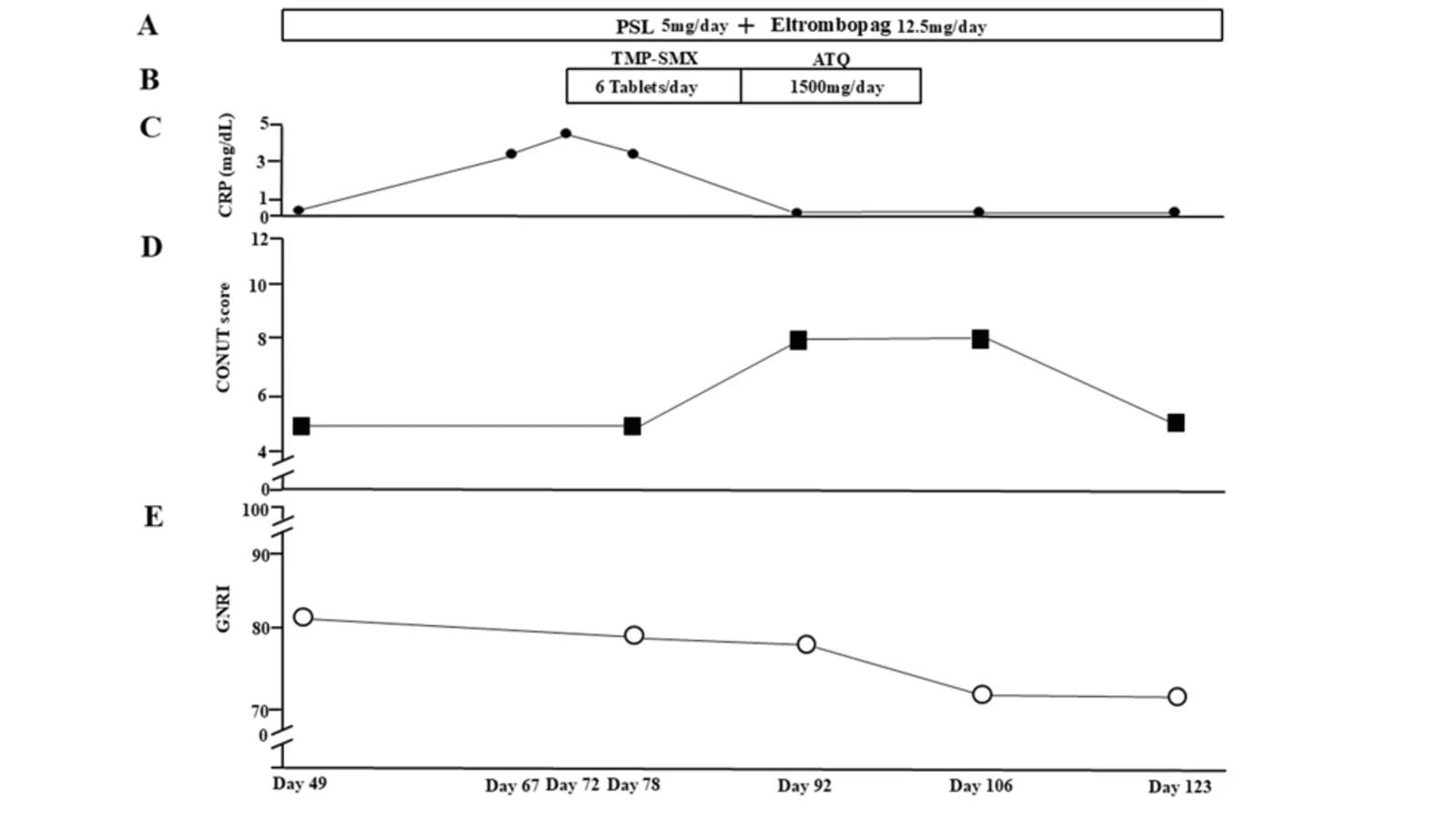
Pharmacologic therapies and serial changes in C-reactive protein levels and nutritional indices. (A) Oral dosage of prednisolone (PSL) and eltrombopag. (B) Oral dosage of trimethoprim-sulfamethoxazole (TMP–SMX; 80 mg trimethoprim/400 mg sulfamethoxazole per tablet) and atovaquone (ATQ). (C) Serial changes in serum C-reactive protein (CRP) levels. (D) Serial changes in the Controlling Nutritional Status (CONUT) score. Malnutrition severity based on the CONUT score was graded as normal (0–1), mild (2–4), moderate (5–8), and severe (9–12). (E) Serial changes in the Geriatric Nutritional Risk Index (GNRI). Nutritional risk according to the GNRI was classified as no risk (>98), low risk (92–98), moderate risk (82–<92), and major risk (<82). The horizontal axis represents the clinical course using day one as the day of hospital admission.

Although serum CRP normalized promptly after initiation of TMP-SMX, a generalized rash appeared on day 83. Because the rash was considered an adverse effect of TMP-SMX, the drug was discontinued, and the patient was switched to atovaquone (ATQ) (Figure 2, Panel B, Figure 3, Panels A, B). The rash resolved shortly thereafter.

Nutritional assessment

Nutritional status was assessed using the CONUT score and the GNRI. The CONUT score was calculated as the sum of the scores for serum albumin, peripheral blood lymphocyte count, and total cholesterol, according to the original CONUT scoring system [12]. Serum albumin was assigned 0, 2, 4, or 6 points for values of ≥3.50, 3.00-3.49, 2.50-2.99, or <2.50 g/dL, respectively; peripheral blood lymphocyte count was assigned 0, 1, 2, or 3 points for ≥1,600, 1,200-1,599, 800-1,199, or <800/μL, respectively; and total cholesterol was assigned 0, 1, 2, or 3 points for ≥180, 140-179, 100-139, or <100 mg/dL, respectively [12]. The GNRI was calculated using the following formula: GNRI = 1.489 × serum albumin (g/L) + 41.7 × (current body weight/ideal body weight). Ideal body weight was estimated using a BMI of 22 kg/m² (ideal body weight (kg) = height (m)² × 22). The patient’s height was 161.8 cm.

Table 1 presents the longitudinal nutritional parameters and the components of each index. Ideal body weight was calculated using a standard height-based formula. The CONUT score was 5 at baseline (day one), increased to 8 during the period of marked nutritional deterioration (day 92), and subsequently improved to 5 by day 123. In contrast, the GNRI declined progressively from 81.3 at baseline to 77.4 on day 92 and 72.8 on day 123, with no evidence of recovery. Thus, the two indices showed divergent longitudinal trajectories despite partial recovery from the acute illness. Figure 3 shows serial changes in serum CRP, CONUT score, and GNRI from Day 49 to Day 123.

**Table 1:** Longitudinal changes in nutrition parameters, CONUT score, and GNRI. Reference values: Serum albumin, 3.8-5.2 g/dL; peripheral blood lymphocytes count, 1,000-4,800/uL; total cholesterol, 150-219 mg/dL. CONUT score was calculated as the sum of the serum albumin score, lymphocyte count score, and total cholesterol score according to the original CONUT scoring system [12]. CONUT score: 0–1, normal; 2–4, mild malnutrition; 5–8, moderate malnutrition; 9–12, severe malnutrition. Alb = serum albumin; Lym = peripheral blood lymphocyte count; Total chol = total cholesterol; BW = body weight; CONUT score = Controlling Nutritional Status score; GNRI = Geriatric Nutritional Risk Index; Ref = reference value

Timeline	Alb (g/dL) (Ref: 3.8–5.2)	Lym (/µL) (Ref: 1,000–4,800)	Total chol (mg/dL) (Ref: 150–219)	BW (kg)	Ideal BW (kg)	CONUT score	GNRI
Day 1	3.0	860	153	50.6	57.6	5	81.3
Day 49	3.0	490	204	50.5	57.6	5	81.2
Day 78	3.0	700	215	47.9	57.6	5	79.3
Day 92	2.9	730	170	47.2	57.6	8	77.4
Day 106	2.5	810	136	49.3	57.6	8	72.9
Day 123	2.9	1,400	201	49.1	57.6	5	72.8

Table 2 presents laboratory findings at admission (day one) and at the diagnosis of probable PCP (day 72).

**Table 2:** Laboratory findings at admission (day one) and at the diagnosis of probable Pneumocystis jirovecii pneumonia (day 72). PLTs = platelets; PA-IgG = platelet-associated immunoglobulin G

Parameter	Value		Reference values
	Day 1	Day 72	
Leukocyte count	6.2 × 10^3^/µL	6.23 × 10^3^/µL	3.5–9.7 × 10^3^/µL
Hemoglobin	9.5 g/dL	12.6 g/dL	12–18 g/dL
PLTs	1.0 × 10^3^/µL	336 × 10^3^/µL	140–379 × 10^3^/µL
PA-IgG	149.2 ng/10^7^ PLTs	Not measured	≤27.6 ng/10^7^ PLTs
Serum albumin	3.0 g/dL	3.0 g/dL	3.8–5.2 g/dL
C-reactive protein	0.64 mg/dL	4.54 mg/dL	0.00–0.30 mg/dL
β-D-glucan	Not measured	171.6 pg/mL	≤20.0 pg/mL
Serum creatinine	0.96 mg/dL	0.77 mg/dL	0.42–0.86 mg/dL

## Discussion

This case demonstrates divergence in the temporal trends of the CONUT score and GNRI in an older patient with multiple comorbidities, including ITP, dementia, and PCP, in the context of decreased oral intake temporally associated with TMP-SMX administration.

Based on the bilateral ground-glass opacities on chest CT and an elevated serum β-D-glucan level, this case was classified as probable PCP, although microbiological confirmation was not obtained. Although a definitive diagnosis was not established, this combination of findings is widely used to diagnose PCP in non-HIV patients [8,14], and the clinical course and treatment response further supported this diagnosis. Other fungal pneumonias, drug-induced pneumonitis, viral pneumonia, and other opportunistic infections were considered in the differential diagnosis; however, given the clinical course, these possibilities were considered unlikely.

A key clinical issue in this case was the marked decrease in oral intake. Although severe appetite loss developed shortly after the initiation of TMP-SMX, this temporal relationship should not be interpreted as evidence of a causal relationship. TMP-SMX has been reported to cause adverse events, including gastrointestinal symptoms and loss of appetite [9,10]. However, reduced food intake in older patients is often multifactorial. In this patient, the underlying probable PCP, systemic inflammation, dementia, hospitalization-related factors, frailty and other underlying conditions, and concomitant medications may all have contributed to the decline in oral intake [11,15]. The subsequent improvement in oral intake after discontinuation of TMP-SMX also does not by itself establish causality, because these other factors changed concurrently during the clinical course. Therefore, the relationship between TMP-SMX administration and reduced oral intake is best considered a temporal association rather than a confirmed drug-related adverse event.

The most notable finding was the longitudinal divergence between the CONUT score and the GNRI. Although the CONUT score initially worsened, it subsequently improved in parallel with recovery from the acute illness (PCP) and partial recovery of serum albumin, lymphocyte count, and serum cholesterol [12]. In contrast, the GNRI showed a persistent decline, primarily reflecting continued weight loss and delayed weight recovery [13]. The discrepancy between the CONUT score and GNRI reflects the different components and clinical characteristics of these indices. The most notable finding was the longitudinal divergence between the CONUT score and the GNRI. The two indices were developed with different components and clinical purposes. CONUT incorporates serum albumin, peripheral blood lymphocyte count, and total cholesterol and was developed for the early detection and continuous monitoring of hospital undernutrition [12]. In contrast, GNRI incorporates serum albumin and the ratio of actual body weight to ideal body weight and was developed to assess nutrition-related risk in older patients, with the original study demonstrating associations with complications and mortality [13]. In the present case, the CONUT score initially increased and subsequently decreased as the values of its laboratory components changed during the clinical course, whereas the GNRI continued to decline as body weight remained below ideal body weight. These observations may explain the divergent trajectories observed in this patient. However, this single case cannot establish that CONUT is inherently more sensitive to short-term changes or that GNRI specifically reflects long-term nutritional status. Rather, the observed divergence should be regarded as a case-based interpretation consistent with the different components and intended clinical applications of the two indices.

This divergence has important clinical implications, particularly for frail older adults. Reliance on a single nutritional indicator may lead to misinterpretation of nutritional recovery, especially when biochemical and immunological improvement precedes weight regain. International guidelines emphasize that nutritional assessment in older patients should be multifaceted [11,15]. Therefore, using both indicators may allow a more comprehensive assessment of nutritional status and the recovery process.

Furthermore, this case underscores the importance of careful nutritional management. The patient temporarily required TPN before oral intake was gradually resumed. Close monitoring of calorie and protein intake, combined with continuous assessment using multiple nutritional indicators, was essential for guiding clinical decisions [11].

This report has several limitations. First, as a single case report, its generalizability is limited. Second, PCP was diagnosed based on clinical and imaging findings without microbiological confirmation, leaving some diagnostic uncertainty. Third, although decreased oral intake was temporally associated with TMP-SMX administration, causality could not be established because infection, systemic inflammation, hospitalization, dementia, frailty and other underlying conditions, and concomitant medications may also have contributed to the decline in oral intake. Therefore, this case should not be interpreted as demonstrating a confirmed causal relationship between TMP-SMX and appetite loss. Finally, because nutritional assessment was limited to the CONUT score and GNRI, the evaluation may not have been sufficiently comprehensive. Larger-scale studies are warranted to validate these findings.

## Conclusions

Longitudinal assessment of the CONUT score and GNRI may provide complementary perspectives on nutritional status in older patients with complex clinical courses. In this case, the two indices showed divergent trajectories, which may have been related, at least in part, to differences in their constituent variables and intended clinical applications. However, this single case does not establish the general clinical utility or superiority of either index, or demonstrate that their combined use improves the detection of malnutrition. Further studies are needed to determine whether similar divergent patterns are consistently observed and whether the two indices may provide complementary information in the longitudinal assessment of nutritional status.
